# Cyclin K and cyclin D1b are oncogenic in myeloma cells

**DOI:** 10.1186/1476-4598-9-103

**Published:** 2010-05-10

**Authors:** Véronique Marsaud, Guergana Tchakarska, Geoffroy Andrieux, Jian-Miao Liu, Doulaye Dembele, Bernard Jost, Joanna Wdzieczak-Bakala, Jack-Michel Renoir, Brigitte Sola

**Affiliations:** 1 Pharmacologie Moléculaire des Anticancéreux, CNRS UMR 8612, IFR 141, Université de Paris-Sud, Châtenay-Malabry, France; 2 Biologie Moléculaire et Cellulaire de la Signalisation, EA 3919, IFR 146, Université de Caen, Caen, France; 3 Institut de Chimie des Substances Naturelles, CNRS UPR 2301, Gif-sur-Yvette, France; 4 Plateforme Biopuces et Séquençage, Institut de Génétique et de Biologie Moléculaire et Cellulaire, Strasbourg, France

## Abstract

**Background:**

Aberrant expression of cyclin D1 is a common feature in multiple myeloma (MM) and always associated with mantle cell lymphoma (MCL). *CCND1 *gene is alternatively spliced to produce two cyclin D1 mRNA isoforms which are translated in two proteins: cyclin D1a and cyclin D1b. Both isoforms are present in MM cell lines and primary cells but their relative role in the tumorigenic process is still elusive.

**Results:**

To test the tumorigenic potential of cyclin D1b *in vivo*, we generated cell clones derived from the non-*CCND1 *expressing MM LP-1 cell line, synthesizing either cyclin D1b or cyclin K, a structural homolog and viral oncogenic form of cyclin D1a. Immunocompromised mice injected *s.c*. with LP-1K or LP-1D1b cells develop tumors at the site of injection. Genome-wide analysis of LP-1-derived cells indicated that several cellular processes were altered by cyclin D1b and/or cyclin K expression such as cell metabolism, signal transduction, regulation of transcription and translation. Importantly, cyclin K and cyclin D1b have no major action on cell cycle or apoptosis regulatory genes. Moreover, they impact differently cell functions. Cyclin K-expressing cells have lost their migration properties and display enhanced clonogenic capacities. Cyclin D1b promotes tumorigenesis through the stimulation of angiogenesis.

**Conclusions:**

Our study indicates that cyclin D1b participates into MM pathogenesis *via *previously unrevealed actions.

## Background

Cyclin D1 is a key actor for the development and progression of various cancers including hematological malignancies. The human *CCND1 *gene generates two mRNA species by alternative splicing [1]. The two corresponding proteins cyclin D1a and D1b differ only in the last 55 amino acids of the carboxy-terminus. Both isoforms possess the N-terminal domain, necessary for retinoblastoma protein (pRb) binding, the cyclin box, required for cyclin-dependent kinase (CDK) binding and activation and the central region, implicated in transcriptional regulation. The PEST sequence which controls protein turn-over and the threonine 286 (Thr286), the site of phosphorylation by glycogen synthase kinase-3β which promotes the nuclear export of cyclin D1 and its degradation through the proteasome pathway [2,3], are present only in cyclin D1a. The oncogenic potential of cyclin D1 seems restricted to the isoform b as shown *in vitro *[4-6]. In transgenic mouse models, inhibition of cyclin D1 proteolysis is the causative factor for mammary carcinomas and B-cell lymphomas [7,8]. The mechanisms of cyclin D1b-mediated tumorigenesis are not fully understood and could depend on the cellular context and in particular on the concomitant expression of cyclin D1a.

Cyclin K is encoded by Kaposi sarcoma-associated herpes virus (KSHV), a human tumor virus associated with the development of Kaposi sarcoma and lymphoid malignancies in immunocompromised individuals, reviewed in [9]. Cyclin K and cyclin D1 share sequence colinearity and identity. The tumorigenic properties of cyclin K have been demonstrated in transgenic animals in which the lymphocyte compartment has been targeted [10]. In a similar transgenic model, cyclin D1a alone fails to induce leukemogenesis [11,12].

Mantle cell lymphoma (MCL) and multiple myeloma (MM) are two hematological malignancies for which cyclin D1 expression has been recognized as an oncogenic event [13,14]. Although cyclin D1a and D1b mRNAs are present in all MCL and MM samples tested, cyclin D1a protein is expressed predominantly [15,16]. However, a role of cyclin D1b in the leukemogenic process cannot be ruled out. In order to study the oncogenic potential of cyclins D1b and K in the context of mature B cells, we generated several cell clones derived from LP-1 MM cell line, expressing either cyclin D1b, Myc or cyclin K oncogenes. LP-1 cell line was chosen because this MM cell line does not express any cyclin D1 isoform. We report here that cyclin D1b- and cyclin K-expressing LP-1 cells are tumorigenic *in vivo *in xenograft models. Genome-wide analysis allowed us to describe several mechanisms for cyclin D1b- and K-mediated oncogenesis.

## Methods

### Generation of LP-1-derived clones

LP-1 MM cell line which does not express cyclin D1 was chosen for the generation of stable transfected clones. GRANTA-519 MCL cell line has the t(11;14)(q13;q32) and expresses high level of cyclin D1a. LP-1 and GRANTA-519 cells were maintained in RPMI 1640 containing 10% fetal calf serum (FCS), L-glutamine and antibiotics (Lonza Verviers SPRL, Verviers, Belgium). pcDNA3-flagged cyclin K [17] (a generous gift of O. Coqueret), pcDNA3-c-Myc (a generous gift of D. Cappellen) and pcDNA3-cyclin D1b [18] encode for the full-length proteins, respectively. LP-1 cells were transfected by electroporation, selected with 500 μg/ml G418, cloned by limiting dilution in 96-well plates. Single clones were individually tested for exogenous protein expression. After three months in culture without loss of transgene expression, G418 was first reduced and finally removed.

### Cell cycle analysis by flow cytometry

Exponentially growing LP-1-derived cells were plated at a density of 5 × 10^5 ^cells/ml, harvested 24 h later, fixed in ice-cold EtOH 80% in PBS. Cells were treated with 100 μg/ml RNase A and 20 μg/ml propidium iodide (PI) for 30 min at 37°C. Cells were analyzed with an Epics XL flow cytometer and data with the Expo™ 32 software (Beckman Coulter, Villepinte, France).

### Matrigel invasion assay

LP-1-derived cells were suspended in FCS-free RPMI 1640 medium and 2 × 10^4 ^cells were placed in the upper chamber of transwell inserts coated with Matrigel (BD BioCoat Matrigel Invasion Chamber, BD Biosciences, Le Pont de Claix, France). In the lower compartment, we added RPMI 1640 medium plus 1% FCS. Plates were incubated for 4 h at 37°C to allow migration of cells. After incubation, inserts were carefully removed, washed, fixed and colored to allow cell counting. Results are expressed as the number of cells that invaded the Matrigel. Statistical analysis between two groups was done with the Student's *t *test.

### Clonogenicity assay

The ability of individual cell to grow in semi-solid support was assayed using MethoCult^® ^(StemCell Technologies, Grenoble, France) according to the manufacturer' instructions. Cells were prepared at a density of 3 × 10^3 ^cells/ml in Iscove's MDM plus 2% FCS; then added to the same volume (3 ml) of methyl cellulose containing phytohemagglutin-leucocyte conditioned medium (PHA-LCM) as source of growth factor. Cells were dispensed in triplicate in Petri dishes, incubated in humidified atmosphere at 37°C for 10 days. Colonies containing more than 50 cells were counted using inverted microscope and gridded scoring dish.

### Immunoblotting

Methods for protein extraction, SDS-PAGE and immunoblotting were described previously [18].

### *In vivo *engraftment experiments

**Figure 1:**
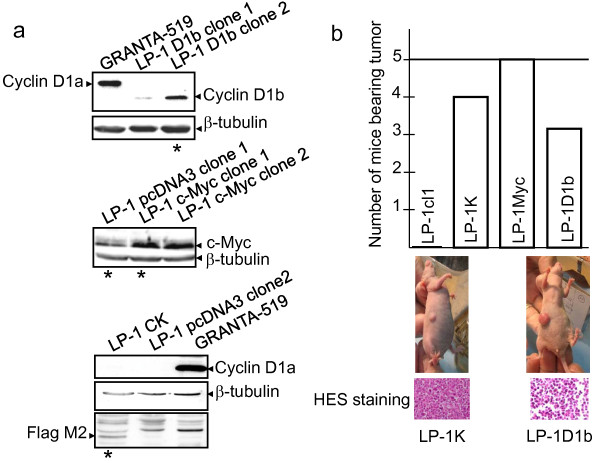
**Cyclin D1b and cyclin K are oncogenic in nude mice**. a) Generation of LP-1-derived clones. Total proteins were extracted from individual clones, resolved by SDS-PAGE (12%) and immunoblotted with anti-cyclin D1 Ab which detects both cyclin D1a and b isoforms (DCS-6, BD Biosciences, Le Pont de Claix, France), anti-c-Myc Ab (sc-764, Santa Cruz Biotech., Santa Cruz, CA, USA), anti-Flag M2 Ab (Sigma-Aldrich, Saint Quentin Fallavier, France) which detects cyclin K construct. Anti-β-tubulin Ab (sc-9104, Santa Cruz biotech.) was used to control gel loading and transfer, GRANTA-519 cell line was used as control for cyclin D1a expression. The four clones then referred as LP-1cl1, LP-1D1b, LP-1 Myc and LP-1K marked with an asterisk (*) were injected *in vivo*. b) Each cell clone was injected with Matrigel *s.c*. in 5 *nude *mice which were sacrificed 8 weeks later. The number of mice with a tumor at the site of injection is presented in the histogram; two representative mice bearing tumor are shown as well as hematoxylin-eosin-safran (HES) staining of tumor sections.

**Figure 2:**
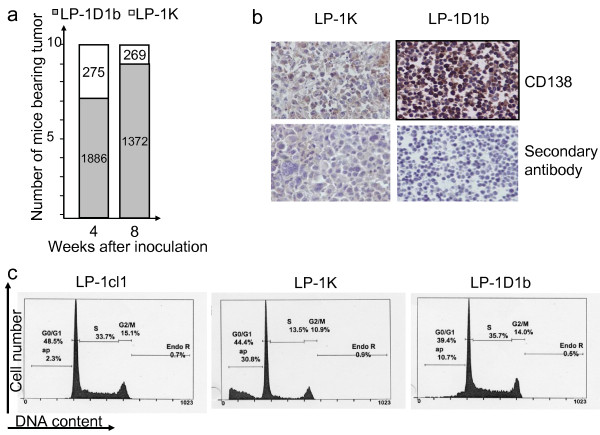
**The engrafment potential of LP-1K and LP-1D1b does not rely on exacerbated proliferation properties**. a) In a second set of engraftment assay, mice were monitored for tumor appearance, and the volume of the tumor evaluated. In the histograms are indicated the number of mice bearing tumors four or eight weeks post-injection and the mean volume of tumors at that time. b) Fixed tumor sections were studied by conventional IHC for CD138 (brown staining) expression (40× magnification). Anti-CD138 Ab was purchased from Dako (Trappes, France). Sequential sections were incubated with the secondary Ab alone as negative control. c) LP-1 derived clones were plated at a density of 5 × 10^5 ^cells/ml, cells were harvested 24 h later, fixed in EtOH, stained with PI and analyzed with an Epics XL flow cytometer and Expo™32 software (Beckman Coulter). For each series, 10,000 to 20,000 events were gated. The percentage of cells within each cell cycle phase (G0/G1, S, G2/M) is indicated on the graph, the apoptotic cells (ap) are in the sub-G1 fraction.

Female, six week-old *nude *mice (NMRI, Janvier, Le Genest Saint-Isle, France), were inoculated *s.c*. with 2.5 × 10^6 ^(1^st ^set) or 4 × 10^6 ^(2^nd ^set) cells of the various clones in Matrigel (BD Biosciences, v/v). Mice were regularly monitored for the development of palpable tumors. Tumor volumes based on caliper measurements were calculated by the ellipsoidal formula [1/2 (length^2 ^× width)]. The first set of animals (five mice per clone) was sacrificed at eight weeks (see Figure 1b). The second series of animals (ten animals per clone) was sacrificed depending on the tumor sizes (see Figure 2a). Tumors were then either fixed in Finefix (Microm Microtech., Francheville, France) or frozen for further analyses. In a third series of experiment, the LP-1D1b clone (5 × 10^6 ^cells) was inoculated in Matrigel into the lower flank of *nude *mice. The day after, 10 μM of either scrambled siRNA (5'-aat tct ccg aac gtg cac gt-3') or siRNA targeting *VEGF *(5'-aag gag acc ctg atg aga tc-3') were mixed with AteloGene™ (Koken, Cosmo Bio Co., Tokyo, Japan) according to manufacturer's instructions. The mixture (150 μl) was *s.c*. injected wrapping up the cells at the injection site. Chemical tyrosine kinase (TK) inhibitors targeting VEGFR2/3 (SAR 131675.13, (SAR)) and all FGFR (SSR 128129E.13, (SSR)), a gift of F. Bono, were dissolved in 5% glucose in physiological serum. SAR and SSR were *i.v*. injected biweekly at 40 mg/kg each, starting at day 1 following inoculation of cells. Each group contained 5 mice. At day 11, volume of tumors was measured as before and the growth of tumors monitored thereafter. The tumor evolution was calculated as the ratio between the volume of tumors at each time point and the volume of the tumors of non treated mice at day 11. Statistical analysis for tumor evolution in each group was done with the Student's *t *test. During the experiments, mice had free access to food and water and all the experiments were performed at the Common Service of Animal Experimentation (UFR de Pharmacie, Châtenay-Malabry), in accordance to the declaration of Helsinki on animal welfare and with the approval of the ethics committee of the University of Paris 11/CNRS (responsible person V. Dommergue-Dupont).

### Immunohistochemistry of tumor sections

Finefix-fixed paraffin embedded 4 μm-sections were deparaffinized in toluene twice for 5 min and rehydrated by using graded EtOH concentrations. After antigen retrieval in citrate buffer pH 6.2 (5 min, 85°C), immunohistochemical labeling with anti-CD138 or anti-CD34 antibodies (Abs) was performed with the Vector Vectastain Elite kit (Vector Laboratories, Burlingame, CA, USA) and 3',3' Diaminobenzidine (DAB) as chromogen. Sections were counterstained with hemalun.

### Microarray hybridization, gene expression data and statistical analyses

For each cell line (LP-1cl1, LP-1K and LP-1D1b), total RNA was extracted from four independent cultures with Trizol reagent (Invitrogen, Cergy Pontoise, France) according to the manufacturer' instructions and used for expression analysis on a 25K human oligonucleotide microarray covering most of the known human transcripts. The 50 mers 5'-amino modified oligonucleotides from the RNG/MRC oligonucleotide collection [19] (information available at http://www.microarray.fr:8080/merge/index) were diluted to a final concentration of 50 mM in 50% dimethyl sulfoxide, 100 mM potassium phosphate (pH 8.0) and printed onto hydrogel-coated slides (Nexterion H slides, Schott, Jena, Germany) using a microGrid II arrayer (Genomic Solutions, Cambridge, UK). Total RNAs (200 ng) were amplified by linear PCR and labelled with Cy3 using Bioprime Array CGH Genomic Labelling System Kit (Invitrogen). Total RNA from one culture of LP-1cl1 cells was similarly amplified, labelled with Cy5 and used as a reference probe for hybridization. Each Cy3-labelled probe was co-hybridized with the Cy5 reference probe on microarrays in a G2545A oven (Agilent, Massy, France) at 60°C for 18 h. Microarrays were washed (10 min in 6× SSC, 0.005% Triton-X100; 5 min in 0.1× SSC, 0.0025% Triton-X100) and scanned with a G2565B scanner (Agilent). Raw data were extracted from scanned microarray images (.tif) using Feature Extraction Software v9.5 (Agilent) and normalized using the Quantile method adapted to bicolour microarrays. All the protocols used can be obtained by contacting the microarray and sequencing platform of the IGBMC (web site: http://www-microarrays.u-strasbg.fr/). In order to select genes that are differentially expressed among the three biological groups (LP-1cl1, LP-1K and LP-1D1b), we performed an analysis of variance using Cy5/Cy3 log2 ratios. To limit the error due to multiple tests, we used permutation of samples for controlling the false discovery rate [20]. Genes with a *p*-value less than 0.01 were considered to be significant. Moreover, we filtered out genes with a fold change (FC). The FC between LP-1K and LP-1cl1 was calculated as the median value of the 4 replicates ratios in the LP-1K samples over the median value of the 4 replicates ratios in the LP-1cl1 samples. Three FC were calculated: LP-1K *vs*. LP-1cl1, LP-1D1b *vs*. LP-1cl1 and LP-1K *vs*. LP-1D1b and a threshold equal to 2 was used for selecting three lists of significant genes. To design Venn diagram, we used the VENNY software http://bioinfogp.cnb.csic.es/tools/venny/ and individual gene expression profiles were generated with the TigrMev 4_03 software http://www.tm4.org/mev.html. To determine functional relationships between genes, we used DAVID Bioinformatics Resources http://david.niaid.nih.gov.

### Real-time quantitative RT-PCR

To validate the microarray data, we used RNAs previously used for microarray hybridization. Primers for *36B4*, *CSN2*, *FGFR3*, *FHIT*, *HSP90B1*, *TUBB2B*, *TFRC*, *CD48*, *LTB*, *FN1*, *BCL2*, *CDK6*, *GAPDH *and *UCHL1 *genes were designed with the LightCycler^® ^Probe Design software (Roche Diagnostics, Meylan, France). Their sequences are reported in the Additional File 1, Table S1. Q-PCR was carried out in a LightCycler^® ^system (Roche Diagnostics) using the LightCycler^® ^FastStart DNA master SYBR Green I kit (Roche Diagnostics) according to the manufacturer's instructions. Cycles were as follows: a 10 min initial cycle at 95°C, followed by 45 cycles of 10 sec of denaturation at 95°C, 5 sec of annealing at 58°C, and 10 sec of extension at 72°C. The specificity of the fluorescence was verified by the melting curve analysis after each reaction. The relative abundance of each target was normalized to *36B4 *expression and the quantification of each mRNA compared to *36B4 *was done using the comparative threshold method (Ct).

### Tumor engraftment onto chick chorio-allantoic membrane

Fertilized chicken eggs (EARL Morizeau, Dangers, France) were handled as described previously [21]. On embryonic day 10, a plastic ring was placed on chick chorio-allantoic membrane (CAM) and 10^7 ^LP-1K or LP-1D1b cells in 30 μl Matrigel (BD Biosciences) were deposited after gentle laceration of the surface. Digital pictures were taken under a stereomicroscope (Nikon SMZ1500) at day 2, 4, 6 of tumor development. Twenty eggs were used for each condition.

## Results

### Cyclin D1b, cyclin K and c-Myc expressing LP-1-derived clones display tumorigenic properties

Stable LP-1 clones were generated by transfection of cyclin D1b-, cyclin K- or c-Myc-expressing pcDNA3 plasmids or empty pcDNA3 as control. As shown Figure 1a, in the two clones LP-1 D1b (1 and 2), the short isoform b of cyclin D1 was expressed (clone 1) or overexpressed (clone 2) at a level comparable to the one in GRANTA-519 MCL cell line which possesses the t(11;14)(q13;q32) and synthesizes high level of cyclin D1a. Endogenous c-Myc was present in the control LP-1 pcDNA3 clone 1, and exogenous c-Myc was overexpressed (×5) in the two LP-1 c-Myc-expressing clones. In the LP-1 CK clone, cyclin K was detected with the anti-Flag M2 Ab. A representative clone from each series (star in Figure 1a), thereafter referred as LP-1cl1 (control), LP-1K, LP-1 Myc or LP-1D1b was injected *s.c*. into a first set of five *nude *mice. Eight weeks after injection, tumors were present at the site of inoculation in 4/5 mice for LP-1K, 5/5 mice for LP-1 Myc and 3/5 mice for LP-1D1b (Figure 1b) but not in mice inoculated with the control clone LP-1cl1. Only one mouse developed a palpable lump (pseudo-tumor, which regresses spontaneously). Macroscopically, tumors were distinguishable from one clone to the other, cyclin D1b-induced tumors being bigger and highly vascularized. After hematoxilin-eosin-safran (HES) staining of fixed tumor sections, histology revealed the presence of typical malignant plasma cells (Figure 1b). In a second series of *in vivo *experiments, 10 animals per cell line were inoculated. Four weeks after injection, tumors were detected at the site of inoculation in 10/10 mice for LP-1K and 6/10 mice for LP-1D1b (Figure 2a). Five mice from each series were sacrificed and the others monitored for four more weeks. At that time, four more mice in the LP-1D1b series bore tumors. The most striking differences between the two series were the size of the tumors (Figure 2a) and again the rich vascularization of LP-1D1b tumors (data not shown). Immunohistological examination of tumor sections indicated that engrafted tumors contained *bona fide *myeloma cells expressing CD138 (Figure 2b). Our data show unambiguously that such as c-Myc, cyclin D1b and cyclin K are capable to confer a malignant phenotype to LP-1 MM cells and are oncogenic *in vivo*.

### Cyclin D1b and cyclin K are not mitogenic in LP-1 cells

We used flow cytometry sorting of PI-stained exponentially growing cells to assess the cell proliferation capacities of LP-1-derived clones. As presented in Figure 2c, the overexpression of cyclin D1b, cyclin K or c-Myc did not enhance the percentage of cells within the S phase of the cell cycle. By contrast, both LP-1D1b and LP-1K exhibited spontaneous apoptosis. In LP-1K cells, we observed a concomitant decrease of DNA synthesizing cells. We concluded from these data that the oncogenic properties acquired by LP-1 cells do not rely on an exacerbated proliferation potential.

### Cyclin D1b and cyclin K expression alter LP-1 cells transcriptome

**Figure 3:**
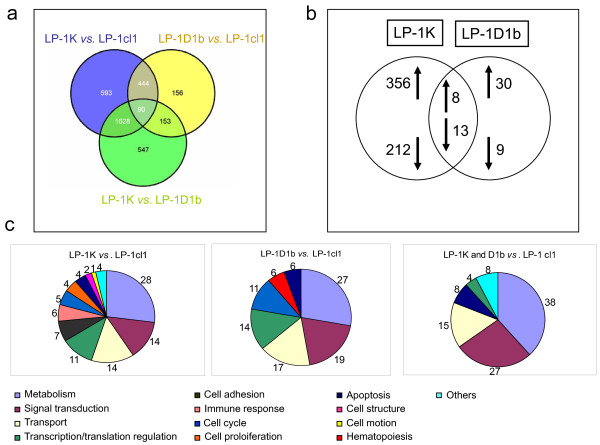
**Transcriptome datasets**. a) The Venn diagram drawn with VENNY software shows the overlaps between the sequences that are the most differentially expressed across the three transcriptome datasets (LP-1K *vs*. LP-1cl1, LP-1D1b *vs*. LP-1cl1 and LP-1K *vs*. LP-1D1b, FC>2). b) We filtered genes coding for proteins involved in biological processes and having a FC>3. We have eliminated from the raw data: doublets, UG clusters corresponding to "data not found", sequences with no gene ontology (GO)-associated terms, non specific terms such as "open reading frame", "hypothetical" and "IMAGE"-containing terms. c) Functions were attributed to genes with DAVID tools. The percentage of altered genes involved in the various cellular functions is indicated by numbers.

We used transcriptome analysis to evaluate cyclin D1b- and cyclin K-induced changes in LP-1 cells. Microarray data and annotations have been deposed in the NIH gene expression Omnibus under accession number GSE15497. A Venn diagram was used to visualize the overlap between three data sets: LP-1K *vs*. LP-1cl1, LP-1D1b *vs*. LP-1cl1, LP-1K *vs*. LP-1D1b (FC>2, Figure 3a). This diagram shows that the expression of cyclin K had major effects on LP-1 transcriptome (593+444+90+1628 sequences were modified); less sequences were altered by both cyclin D1b and cyclin K (444+90) or cyclin D1b alone (156+153). We then filtered sequences to select genes coding for proteins having known biological functions and FC>3 to limit the number of genes to study. The number of genes up- or down-regulated in LP-1K or/and LP-1D1b cells is indicated in Figure 3b. Individual gene expression profiles were generated with the TigrMev 4_03 software (Additional File 2 Figure S1, Additional File 3 Figure S2 and Additional File 4 Figure S3). We then hierarchically clustered genes on the basis of their biological processes (Figure 3c). Numerous genes implicated in metabolism, signal transduction, transport, transcriptional and translational regulations were modified by cyclin K and/or cyclin D1b. Unexpectedly, genes regulating cell cycle, apoptosis, cell proliferation were less numerous. Genes involved in cell structure and cell motion were specifically modified by cyclin K, whereas genes regulating hematopoiesis were modified by cyclin D1b. Our data indicate that the transformation process elicited by cyclin D1b and cyclin K involved a broad range of cellular processes.

### Cyclin D1b and cyclin K alter cell cycle and survival genes expression

**Figure 4:**
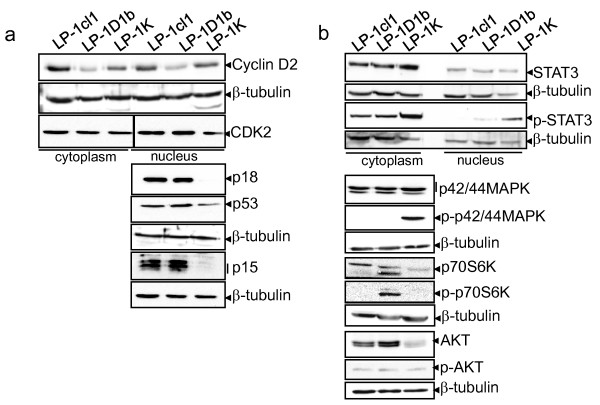
**Cyclin K and cyclin D1b impact the biology of LP-1 cells**. Proteins from exponentially growing cells were resolved by SDS-PAGE and immunoblotted with the following Abs: anti-cyclin D2 (sc-181), anti-CDK2 (sc-6248), anti-p15 (sc-612), anti-p18 (sc-865), anti-β-tubulin (sc-9104) from Santa Cruz Biotech.; anti-p53 (Ab-1, Calbiochem, Merck Chemicals Ltd., Nottingham, UK); anti-p44/42 MAPK (#9102), anti-phospho-p44/42 MAPK (Thr202/Tyr204) (#9101), anti-p70S6K (#9202), anti-phospho-p70S6K (Thr389) (#9205), anti-AKT (#9272), anti-phospho-AKT (Thr308) (#4055), anti-Stat3 (#9132), anti-phospho-Stat3 (Ser727) (#9134, Cell Signaling Technology, Danvers, MA, USA). Blots were reprobed with an anti-β-tubulin Ab as control of charge and transfer.

Real-time RT-PCR was performed for validation of microarray results (Table 1). We found a good correlation between microarray and RT-PCR data for the altered expression of 7 genes in LP-1D1b and 6 genes in LP-1K. Western blots, flow cytometry (data not shown) and immunocytochemical assays further confirmed transcriptional data (Figure 4a, b). Among the genes encoding cell cycle-associated proteins altered in LP-1 derivatives (Table 2 and data not shown), we confirmed the downregulation of cyclin D2 in LP-1D1b cells (FC: -2.05), the downregulation of CDK2 in LP-1K cells (FC: -2.10), the complete disappearance of p18^INK4C ^in LP-1K cells, a clear decrease of p53 level in LP-1K cells (Figure 4a). Although the level of transcription of the *TP53 *gene itself was not modified in LP-1K *vs*. LP-1cl1 cells, the transcription of two genes coding for two proteins involved in p53 stabilization were downregulated. These two proteins are the tumor protein p53 inducible protein 3 (*TP53I3*, FC: -3.57) and binding protein 2 (*TP53BP2*, FC: -2.12). *CDKN2B *mRNA was decreased both in LP-1K and LP-1D1b cells. However, at the protein level, no major differences were seen between LP-1cl1 and LP-1D1b whereas p15^INK4B ^disappeared totally in LP-1K cells (Figure 4a). Differences of post-transcriptional mechanisms in each cell line could explain this variation between microarray and western blot data.

**Table 1:** Real-time quantitative RT-PCR for validation of microarray data

Gene	Microarray data	ΔCt (Ct LP-1D1b-Ct LP-1cl1)	Fold change	FC (microarray)
*36B4*	nm*	-0.78/-0.52/-0.42**	1	**-**
*CSN2*	+7.33	-2.16/-2.70	+7.67/+6.34	**+7.33**
*FGFR3*	+5.07	-2.78/-1.62	+11.79/+4.25	**+5.09**
*FHIT*	+4.67	-1.41/-1.10	+3.68/+3.68	**+4.67**
*HSP90B1*	+2.25	-1.21	+3.20	**+2.26**
*TUBB2B*	-2.32	1.01	-1.45	**-2.32**
*TFRC*	-6.48	3.77/3.14	-9.51/-6.36	**-6.48**
*CD48*	-12.27	4.05/4.18	-9.64/-13.08	**-12.27**

**Gene**	**Microarray data**	**ΔCt (Ct LP-1K- Ct LP-1cl1)**	**Fold change**	**FC (microarray)**

*36B4*	nm*	-0.25/-0.40/-0.20/-0.36	1	**-**
*LTB*	+40.66	-5.7	+43.71	**+40.67**
*FN1*	+13.54	-3.55	+8.87	**+13.54**
*BCL2*	+3.17	-2.36	+3.89	**+3.17**
*CDK6*	-4.14	1.83/1.95	-4.08/-4.43	**-4.14**
*GAPDH*	-5.15	1.09	-2.44	**-6.15**
*UCHL1*	-63.82	11.11/11.25	-2538/-2797	**-63.82**

* nm, not modified. **When several numbers are indicated, they refer to the results obtained with different runs of PCR. For each sample, the average Ct value for the internal standard *36B4 *was subtracted from the average Ct value for each gene to yield ΔCt. The relative amount of each mRNA compared to the calibrator (*36B4*) in each run was calculated by the formula N = 2^-ΔΔCt ^to give the fold change. For each gene, the Fc calculated from microarray data (in bold) is reported in the right column.

**Table 2:** Genes coding for cell cycle regulatory molecules displaying altered expression in LP-1 derivatives (|FC|>3)

Gene	Protein	LP-1D1b *vs*. cl1	LP-1K *vs*. cl1
*SESN2*	Sestrin 2	+3.36*	nm**
*DDIT3*	DNA-damage-inducible transcript 3	+3.35	nm

*CCNB1IP1*	Cyclin B1 interacting protein 1	nm	+10.36
*RASSF5*	Ras association (RalGDS/AP-6) domain family member 5	nm	+4.79
*CDNK1A*	Cyclin-dependent kinase inhibitor 1A	nm	+4.30
*CABLES1*	CDK5 and ABL enzyme substract 1	nm	+3.30
*MAD2L1*	MAD2 mitotic arrest deficient-like 1	nm	-3.13
*CCNB2*	Cyclin B2	nm	-3.14
*GAS2*	Growth-arrest specific 2	nm	-3.22
*MK167*	Antigen identified by monoclonal antibody Ki67	nm	-3.37
*PINX1*	PIN2-interacting protein 1	nm	-3.57
*CCNF*	Cyclin F	nm	-3.65
*CKS2*	CDC28 protein kinase regulatory subunit 2	nm	-3.68
*CHMP1A*	Chromatin modifying protein 1A	nm	-3.87
*CDK6*	Cyclin-dependent kinase 6	nm	-4.14
*CCNB1*	Cyclin B1	nm	-5.23
*CDKN3*	Cyclin-dependent kinase inhibitor 3	nm	-6.53
*CDKN2C*	Cyclin-dependent kinase inhibitor 2C (p18)	nm	-8.18

*CDKN2B*	Cyclin-dependent kinase inhibitor 2B (p15)	-3.75	-2.83

* Numbers are the fold change of the sample compared to LP-1cl1; ** nm, not modified.

Then, we analyzed the status of signalization pathways in LP-1 cells. Indeed, microarray data indicated that either signalization from transmembrane receptors (epithelial growth factor receptor (EGFR), tumor necrosis factor receptor (TNFR), hepatocyte growth factor receptor (HGFR), interleukin-21 receptor (IL-21R) etc.) or signalization molecules belonging to the phosphoinositol-3 kinase (PI3K)/AKT, Janus kinase (JAK)/signal transducer and activator of transcription 3 (STAT3), mitogen-activated protein kinase (MAPK), nuclear factor (NF)-κB could be altered in LP-1 derived cells (Table 3). This was verified by immunoblotting (Figure 4b). The STAT3 pathway is constitutively activated in LP-1 cells. In LP-1K cells, this pathway is overactivated as shown by the hyperphosphorylation of STAT3 both in the cytoplasmic and nuclear compartments. The MAPK pathway is activated in LP-1K cells whereas the p70S6K pathway is activated in LP-1D1b cells. The AKT protein is downregulated in LP-1K cells. These data underline that, although structurally related, cyclin D1b and cyclin K are able to activate/inhibit different signaling pathways controlling survival and/or proliferation.

**Table 3:** Genes coding for signalization molecules displaying altered expression in LP-1 derivatives (|FC|>3)

Gene	Protein	LP-1D1b *vs*. cl1	LP-1K *vs*. cl1
*FGFR3*	Fibroblast growth factor receptor 3	+5.08*	nm**

*AKT3*	v-akt oncogene homolog 3	nm	-5.16
*MET*	HGFR, Met proto-oncogene	nm	-4.33
*ITPKA*	Inositol 1,4,5-triphosphate 3-kinase A	nm	-3.94
*CD81*	CD81 molecule	nm	+3.03
*PIK3CG*	Phosphoinositide 3-kinase gamma	nm	+3.20
*DOK6*	Docking protein 6	nm	+3.21
*MAPK13*	MAP kinase 13	nm	+3.25
*ECOP*	EGFR-overexpressed protein	nm	+3.39
*PRKD2*	Protein kinase D2	nm	+3.94
*DUSP6*	Dual specificity phosphatase 6	nm	+8.37
*SKAP1*	Src kinase associated phosphoprotein	nm	+9.28
*SYK*	Spleen tyrosine kinase	nm	+9.87
*MAPK12*	MAP kinase 12	nm	+10.10

*BLK*	B lymphoid tyrosine kinase	+2.39	+12.25

*, ** see legend of Table 2.

The large number of genes and pathways altered by cyclin D1b and/or cyclin K expression precludes a thorough analysis in this manuscript. We focused on two discrete functions of cyclins D-type identified by the microarray analysis and well-known as support for tumorigenic process: cell migration and angiogenesis.

### Cyclin K inhibits migration of LP-1-derived clones and enhances its clonogenic capacities

**Figure 5:**
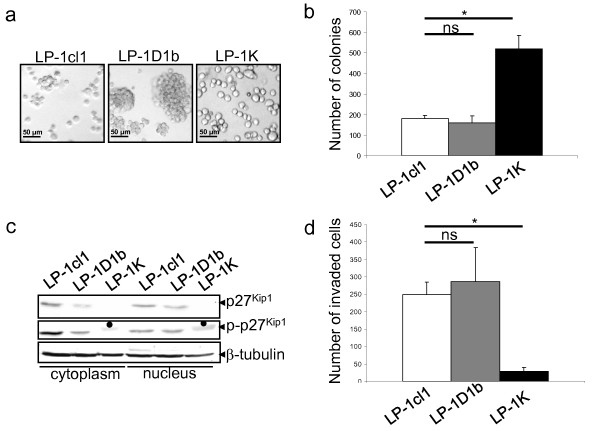
**Cyclin D1b- and cyclin K-expressing LP-1 cells display opposite clonogenic and migration properties**. a) Exponentially growing cells were observed with an inverted phase contrast microscope and photographed. b) Clonogenic assay of LP-1-derived clones. Cells were prepared at a density of 3 × 10^3 ^cells/ml in MethoCult^® ^containing PHA-LCM as source of growth factor (StemCell Technologies). Cells were dispensed in triplicate in Petri dishes, incubated in humidified atmosphere at 37°C for 10 days. Colonies containing more than 50 cells were counted using inverted microscope and gridded scoring dish. Each experiment was done in triplicate and repeated thrice. Results are expressed as mean ± SD. * *p *< 0.05 with the Student's *t *test. c) Western blot analysis of LP-1-derived clones. Either cytoplasmic or nuclear extracts were prepared, separated by 12% SDS-PAGE. Blots were then sequentially incubated with anti-p27^Kip1 ^(sc-528), anti-phospho-p27^Kip1 ^(sc-9104 from Santa Cruz Biotech.) Abs and anti-β-tubulin Ab to control gel loading and transfer. In the cytosolic and nuclear extracts from LP-1K cells, the anti-phospho-p27^Kip1 ^Ab reveals a band which is not at the expected size and likely represents a non specific binding (black dot). d) Migration assay of LP-1-derived clones. SVF (1%) was placed in the lower chamber of a Matrigel-coated transwell, LP-1 cells were plated (2 × 10^4 ^cells) in the upper chamber, incubated 4 h at 37°C. After incubation, invading cells were fixed, stained and counted. Each experiment was done in triplicate and repeated thrice. Results are expressed as mean ± SD. * *p *< 0.05 with the Student's *t *test.

When observed with an inverted optical microscope, LP-1-derived clones exhibited different morphologies (Figure 5a). Compared to LP-1cl1 cells, LP-1D1b formed clusters of cells whereas LP-1K cells grew individually. At the transcriptional level, LP-1K but not LP-1D1b cells displayed major alterations of genes coding for attachment proteins such as integrins, lamin B, ADAMs, ICAMs, CD47 (Table 4). Explaining new morphological properties of the cells, we found that the gene *ITGB7 *coding for integrin β7, recognized as a major promoter of MM cell proliferation trough interactions with stroma cells [22] was downregulated in LP-1D1b cells and upregulated in LP-1K cells. LP-1K cells showed enhanced clonogenic capacities when plated in semi-solid medium compared to LP-1cl1 and LP-1D1b which showed similar capacities (Figure 5b). Cyclin D1 regulates cell proliferation and cell migration of mammary epithelial cells through the stabilization of p27^Kip1 ^and its phosphorylation of a Ser10 residue [23]. We analyzed the level and the phosphorylated status of p27^Kip1 ^in LP-1-derived cell clones (Figure 5c). Both the levels of p27^Kip1 ^protein and its phosphorylated form were lower in LP-1D1b cells than in LP-1cl1 and p27^Kip1 ^was no longer expressed in LP-1K cells both in the nuclear and cytoplasmic compartments. These results argue that cyclins D1b and K fail to stabilize p27^Kip1^. We next studied the migration properties of LP-1-derived clones by the Matrigel invasion assay. Compared to LP-1cl1 cells, LP-1D1b had a similar capacity to migrate whereas LP-1K cells had completely lost this migratory property (Figure 5d).

**Table 4:** Genes coding for molecules controlling adhesion and movement displaying altered expression in LP-1 derivatives (|FC|>3).

Gene	Protein	LP-1D1b *vs*. cl1	LP-1K *vs*. cl1
*CX3CR1*	Chemokine (C-X-C motif) receptor 1	nm*	+63.24**
*CD99*	CD99 molecule	nm	+52.47
*FXYD5*	FXY domain containing ion transport regulator 5	nm	+43.74
*CD9*	CD9 molecule	nm	+35.80
*SPON1*	Spondin 1	nm	+21.37
*CD4*	CD4 molecule	nm	+18.57
*CXCL12*	Chemokine (C-X-C motif) ligand 12	nm	+17.26
*ARGDIB*	RhoGDP dissociation inhibitor (GDI) beta	nm	+13.55
*FN1*	Fibronectin 1	nm	+13.54
*SUT3*	SUT homolog 3	nm	+13.44
*ICAM3*	Intracellular adhesion molecule 3	nm	+13.34
*LAMB3*	Laminin beta 3	nm	+12.74
*PCDH1*	Protocadherin 1	nm	+12.05
*CNTNAP2*	Contactin associated protein-like 2	nm	+11.79
*MCAM*	Melanoma cell adhesion molecule	nm	+10.70
*CCL2*	Chemokine (C-C motif) ligand 2	nm	+10.09
*SYK*	Spleen tyrosine kinase	nm	+9.87
*ANXA9*	Annexin A9	nm	+9.33
*LAMA3*	Laminin alpha 3	nm	+8.46
*LPXN*	Leupaxin	nm	+7.86
*CD93*	CD93 molecule	nm	+7.60
*ERBB2IP*	Erbb2 interacting protein	nm	+6.12
*NRCAM*	Neuronal cell adhesion molecule	nm	+6.09
*ITGB2*	Integrin beta 2	nm	+6.08
*PCDH1*	Protocadherin 1	nm	+5.77
*CD97*	CD97 molecule	nm	+5.38
*ADAM23*	ADAM metallopeptidase domain 23	nm	+4.90
*CTGF*	Connective tissue growth factor	nm	+4.60
*CD36*	CD36 molecule	nm	+4.53
*NLGN1*	Neuroligin	nm	+4.50
*CD44*	CD 44 molecule	nm	+4.21
*CNTNAP2*	Contactin associated protein-like 2	nm	+4.03
*CD33*	CD33 molecule	nm	+4.02
*HNT*	Neurtrimin	nm	+3.97
*SELPLG*	Selectin P ligand	nm	+3.92
*PKD2*	Polycystic kidney disease	nm	+3.80
*SIGLEC7*	Sialic acid binding Ig-like lectin 7	nm	+3.56
*L1CAM*	L1 cell adhesion molecule	nm	+3.21
*COL18A1*	Collagen type XVIII alpha 1	nm	+3.20
*ADAM15*	ADAM metallopeptidase domain 15	nm	+3.18
*CDSN*	Corneodesmosin	nm	+3.17
*SIGLEC9*	Sialic acid binding Ig-like lectin 9	nm	+3.11
*NEO1*	Neogenin homolog 1	nm	-3.22
*TROAP*	Trophilin-associated protein	nm	-3.60
*ITGA6*	Integrin alpha 6	nm	-3.86
*ITGAE*	Integrin alpha E	nm	-3.96
*DST*	Distonin	nm	-4.00
*COL24A1*	Collagen type XXIV alpha 1	nm	-4.07
*JAM3*	Junctional adhesion molecule 3	nm	-4.26
*PKP2*	Plakophilin 2	nm	-5.11
*SPN*	Sialophorin (CD43)	nm	-5.55
*JAM2*	Junctional adhesion molecule 2	nm	-12.09

*ITGB7*	Integrin beta7	-4.26	+4.65

*, ** see legend of Table 2.

### Cyclin D1b allows neo-angiogenesis of engrafted tumors

**Figure 6:**
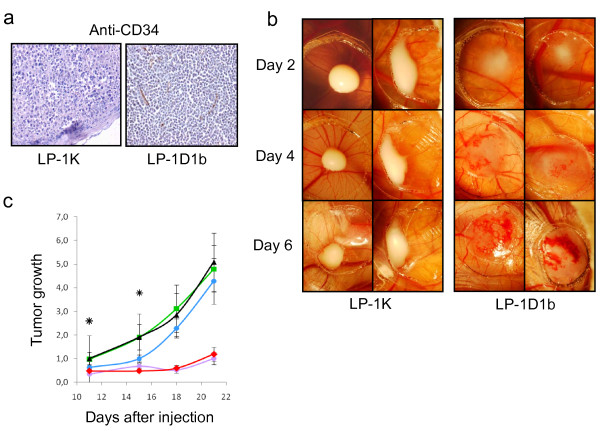
**Cyclin D1b promotes tumor growth by stimulating angiogenesis**. a) Fixed tumor sections were studied with conventional IHC for CD34 (brown staining) expression. Anti-CD34 Ab (MEC14.7) was purchased from Hycult Biotech. (Uden, The Netherlands). Images (40× magnification) are representative of 3 experiments performed on 3 different tumors. b) Fertile eggs were incubated at 37.8°C and 80% humidity. On day 10, LP-1K and LP-1D1b cells (2 × 10^7 ^cells per egg) were inoculated on the CAM. At days 2, 4 and 6 after cells implantation, digital images of primary tumors were acquired at ×7.5 magnification. c) Five mice per condition were inoculated *s.c*. with LP-1D1b cells. For siRNA experiments, mice were injected the day after with a mixture of AteloGene™ and scrambled (green square) or VEGF (blue circle) siRNA. For chemical inhibitors experiments, mice were injected biweekly starting at day 1 following cell injection with SSR (purple circle) or SAR (red diamond). Non treated mice were used as controls (black triangle). The volume of tumors was measured at day 11 and during the period indicated and the tumor growth evaluated. The groups of mice injected with VEGF siRNA and untreated were compared at days 11 and 15; *, *p *< 0.05.

LP-1 cells such as myeloma cell lines synthesize angiogenic factors such as vascular endothelial growth factor (VEGF) (data not shown). Cyclin D1b and/or cyclin K expression in LP-1 cells impacted on proangiogenic and antiangiogenic gene expression (Table 5). Compared with LP-1K-, LP-1D1b-derived tumors were highly vascularized (Figure 1b). This was confirmed by IHC after labeling the CD34 antigen present on vessel endothelial cells. As observed in Figure 6a, CD34 staining is detected mainly in LP-1D1b-derived tumors. The CAM assay was performed to evaluate the direct effect of cyclins D1b and K on tumor engraftment and tumor-mediated angiogenesis. Both cyclin D1b- and cyclin K-expressing LP-1 cells were able to generate tumors in the CAM model within few days. As shown in Figure 6b, LP-1D1b cells gave rise to evolutive tumors characterized by higher volume and significantly greater vascularization than LP-1K cells. Tortuous capillaries are visible at the surface of LP-1D1b tumors while LP-1K tumors, characterized by lack of size progress, were not perfused. Thus, cyclin D1b promotes neoangiogenesis and consequently, tumor growth *in vivo*. To confirm the involvement of neoangiogenesis in tumorigenesis of LP-1D1b cells in xenografts, we injected either once VEGF siRNA (or the control scrambled siRNA) at the vicinity of the injection site or biweekly, chemical FGFR or VEGFR inhibitors, SSR and SAR respectively. As shown Figure 6c, as expected, scrambled siRNA had no effects on tumor evolution. Administration of VEGF siRNA markedly diminished the volume of LP-1D1b-derived tumors for a 15 day-period. After 15 days, no more effects of VEGF siRNA were observed likely due to siRNA degradation and the tumor grew with a rate similar to the one of control. This is in agreement with the reported stability of siRNA in the delivery gel [24]. Importantly, SSR and SAR inhibitors completely abolished the growth of tumors indicating a role of FGFR and VEGFR in the tumor evolution. The capacity of VEGF siRNA as well as TK inhibitors to inhibit tumor growth strongly supports microarray and CAM data and the conclusion that cyclin D1b favors tumorigenesis through activation of a neoangiogenic process.

**Table 5:** Genes coding for proangiogenic or antiangiogenic molecules displaying altered expression in LP-1 derivatives (|FC|>3).

Gene	Protein	LP-1D1b *vs*. cl1	LP-1K *vs*. cl1
*FGFR3*	Fibroblast growth factor receptor 3	+5.08*	nm**
*TNFRSF10B*	Tumor necrosis factor receptor superfamily member 10 B	+3.52	nm
*GATA4*	GATA binding protein 4	+2.15	nm
***WARS***	**Tryptophanyl-tRNA synthase**	**+4.01**	**nm**
*IGFBP3*	Insulin-like growth factor binding protein 3	nm	+26.42

*EPAS1*	Endothelial PAS domain protein 1	nm	+17.39
*CXCL12*	CXC chemokine ligand 12	nm	+17.26
*SLIT3*	SLIT homolog 3	nm	+13.44
*CCL2*	Chemokine ligand 2	nm	+10.09
*CXCL16*	CXC chemokine ligand 16	nm	+6.67
*F11R*	F11 receptor	nm	+5.56
*RUNX1*	Runt-related transcription factor 1	nm	+5.13
*EGFL7*	EGF-like domain multiple 7	nm	+4.68
*CTGF*	Connective tissue growth factor	nm	+4.60
*TNFSF13*	Tumor necrosis factor superfamily member 13	nm	+4.57
*SERPINB6*	Serpin peptidase inhibitor clade B member 6	nm	+4.53
*PDGFB*	Platelet-derived growth factor beta	nm	+3.99
*CXCL3*	CXC chemokine ligand 3	nm	+3.76
*SERPINE2*	Serpin peptidase inhibitor clade E member 2	nm	+3.40
*SERPINB1*	Serpin peptidase inhibitor clade B member 1	nm	+3.36
*IL8*	Interleukin 8	nm	+3.30
*MMP13*	Matrix metallopeptidase 13	nm	+3.12
*IFNGR1*	Interferon gamma receptor 1	nm	+3.06
*HAND2*	Heart and neural crest derivatives	nm	-3.04
*NRP2*	Neuropilin	nm	-3.25
*PTPRF*	Protein tyrosine phosphatase receptor type F	nm	-4.20
*RUNX1T1*	Runt-related transcription factor 1 translocated to 1	nm	-4.79
*ADM*	Adrenomedullin	nm	-6.66
*ID1*	Inhibitor of DNA binding 1	nm	-8.32
***LAMA3***	**Laminin alpha 3**	**nm**	**+8.45**
***IFI16***	**Interferon gamma-inducible protein 16**	**nm**	**+4.96**
***JAG2***	**Jagged 2**	**nm**	**+4.77**
***ZFP36***	**Zinc finger protein 36**	**nm**	**+3.60**
***ZFP36L2***	**Zinc finger protein 36 C3H type-like 2**	**nm**	**+3.60**
***BMPR1A***	**Bone morphogenetic protein receptor type 1A**	**nm**	**-3.65**

*PTPRM*	Protein tyrosine phosphatase receprot type M	+2.11	+10.38
*JDB2*	Jun dimerization protein 2	+3.70	+3.74
*IGF2BP1*	Insulin-like growth factor 2 binding protein 1	-2.52	-5.70
***COL9A1***	**Collagen type IX alpha 1**	**-3.41**	**-4.87**
***COL24A1***	**Collagen type XXIV alpha 1**	**-2.42**	**-4.07**
***DAPK1***	**Death-associated protein kinase 1**	**+2.48**	**-3.06**

*, ** see legend of Table 2. Proangiogenic and antiangiogenic (in bold) genes are indicated.

## Discussion

Cyclin D1 is overexpressed in a broad range of solid malignancies, expressed in lymphoid tumors such as MM and MCL and not in their normal counterparts. However, *in vivo *studies failed to reveal a strong oncogenic potential of the conventional cyclin D1, referred to cyclin D1a [11,12]. By contrast, the cyclin D1 isoform b and the mutant cyclin D1 T286A are capable to transform cells *in vitro *[4-6] and to induce tumors *in vivo *[7,8]. These two forms of cyclin D1 share a strict nuclear localization suggesting that nuclear functions of cyclin D1 are necessary and/or sufficient for tumor formation. Mutations of the *CCND1 *gene disrupting the phosphorylation at Thr286 and thereby leading to nuclear accumulation of cyclin D1 have been described in endometrial and esophageal carcinomas further reinforcing this notion [25,26]. However, the molecular mechanisms of cyclin D1b-driven tumorigenesis are not fully elucidated. In cultured cells, cyclin D1b is not capable to activate its catalytic partner CDK4 and in turn, does not regulate positively the cell cycle [5,18], retains a strong transcriptional co-repressor activity, displays reduced binding to p27^Kip1 ^and does not control cell migration [23]. Here we show that, in the context of MM cells, cyclin D1b confers a full malignant phenotype and allows cells engraftment in immune-compromised mice. The genome-wide analysis of LP-1D1b cells extends our understanding of the biological properties of cyclin D1b. Moreover, we have identified genes regulated by cyclin K, a viral oncogenic homolog of cyclin D1a and confirm the fundamental differences between the two cyclin D1 isoforms.

### Cyclin D1b and cyclin K alter LP-1 cells metabolism

The tumorigenic properties of cyclins D1b and K are not conferred by an exacerbated proliferation. LP-1D1b and LP-1K cells display the same proliferation properties and cyclin D1b or cyclin K expressions have no major impact on cell cycle regulation. Conversely, genes involved in metabolism, signal transduction, transport, transcriptional and translational regulations are profoundly altered by cyclin D1b and/or cyclin K. *In vivo*, cyclin D1 inhibits oxidative glycolysis, lipogenesis, and mitochondrial gene activity in the mammary epithelium [27,28]. In both LP-1K and LP-1D1b cells, the gene transcription of *LDHA *(lactate dehydrogenase, FC: -4.37 and -10.78, respectively), *GAPDH *(glyceraldehyde-3-phosphate dehydrogenase, FC: -4.94 and -3.17, respectively) and *ALDOA *(aldolase A, FC: -2.69 and -3.73, respectively) is decreased. These enzymes catalyze important energy-yielding steps in carbohydrate metabolism. The expression of genes coding for key enzymes involved in oxidative glycolysis such as pyruvate kinase (*PKM2*, FC: -3.57), phosphoglycerate kinase 1 (*PGK1*, FC: -2.10), enolase 1 (*ENO1*, FC: -2.32) in LP-1D1b cells; enolase 2 (*ENO2*, FC: -2.82) in LP-1K cells are down-regulated. This suggests a reduction of glycolysis in tumor cells and, therefore, such as in mammary tumor cells, a paradoxical role of cyclin D1 [27]. Indeed, most of tumor cells show an enhanced glycolytic flux [29]. However, only fast growing tumor cells display markedly modified energy metabolism and multiple myeloma cells are considered as accumulating cells rather than proliferating cells.

### Cyclin D1b and cyclin K modulate gene transcription and translation within LP-1 cells

The roles of cyclin D1 in regulating signal transduction, transcription and translation and their relevance in the cellular transformation process are documented [30]. Among the candidate effectors of cyclin D1 in cancer cells is the transcription factor C/EBPβ [31]. It has been shown, in breast cancer cells, that C/EBPβ is a constitutive repressor of cyclin D1 target genes and that cyclin D1 acts by antagonizing this repressor function. The disruption of signaling through C/EBPβ contributes to breast cell transformation. Interestingly, in LP-1D1b cells, we noticed the up-regulation of *CEBPG *(FC: +3.08), coding for a close related transcription factor C/EBPγ whose function in myeloma cells remains to be established. But it is tempting to speculate some functional redundancy between the two factors. Among the transcription factors altered by cyclin K and/or cyclin D1b expressions, besides MYC, MAF, ETS family members, well-known as major oncogenic actors in plasma cells [13], several others have been implicated in myeloma pathology such as ATF3/4, IRF4/8, NOTCH2, RUNX1/2, XBP1 through the modulation of genes controlling growth, survival and migration. All of them are altered in LP-1K and/or LP-1D1b cells (Table 6). In good correlation, survival and proliferation properties of LP-1K and LP-1D1b cells are modified compared with LP-1cl1 cells.

**Table 6:** Genes coding for transcription factors displaying altered expression in LP-1 derivatives.

Gene	Protein	LP-1D1b *vs*. cl1	LP-1K *vs*. cl1
*DDIT3*	DNA-damage-inducible transcript 3	+3.34*	nm**
*ATF3*	Activating transcription factor 3	+2.27	nm
*XBP1*	X-box binding protein 1	+2.24	nm
*IRF8*	Interferon regulatory factor 8	+2.21	nm

*RUNX1*	Runt-related transcription factor 1	nm	+5.13
*TCF4*	Transcription factor 4	nm	+4.40
*MITF*	Microphtalmia-associated transcription factor	nm	+3.50
*NOTCH2*	Notch homolog 2	nm	+3.36
*STAT5A*	Signal transduction and transcription factor 5A	nm	+2.49
*ETV6*	Ets variant 6	nm	+2.32
*MAF*	v-maf musculoaponeurotic fibrosarcomaa oncogene homolog	nm	+2.25
*MYC*	v-myc myelocytomatosis viral oncogene homolog	nm	+2.08
*ETS2*	v-ets erythroblastosis virus E26 oncogene homolog	nm	+2.06
*IRF4*	Interferon regulated factor 4	nm	-2.25
*RBPJ*	Recombination signal binding protein for Ig kappa region	nm	-2.38
*REL*	v-rel reticuloendotheliosis viral onvogene homolog	nm	-3.63
*RUNX2*	Runt-related transcription factor related 2	nm	-4.80
*MYBL2*	v-myb myeloblastosis viral oncogene homolog-like 2	nm	-5.18

*ATF4*	Activating transcription factor 4	+2.75	+2.64

*, ** see legend of table 2. Since minor alterations in the level of transcription factors could have dramatic effects on gene expression, we sorted genes with a |FC|>2. Only genes coding for transcription factors recognized in the literature as relevant for myeloma pathogenesis are indicated.

Eukaryotic initiation factors (eIFs) control translation at the limiting step of initiation and several of them have been recognized as major actors in transformation processes [32]. In LP-1D1b cells, several genes coding for eIFs are upregulated (*EIF4EBP1*, FC: +3.35; *EIF3EIP*, FC: +2.79; *EIF4A2*, FC: +2.50; *EIF3F*, FC: +2.05; *EIF1*, FC: +2.02). By contrast, in LP-1K, EIF3A and EIF5 are downregulated (FC: -2.28 and -2.92, respectively). A more active translation likely explains the faster growth of LP-1D1b-derived tumors compared to LP-1K tumors.

### Cyclin D1b and cyclin K have opposite action on LP-1 cells migration

Clinical observations indicate that cyclin D1 overexpression in human cancers correlate with metastasis. In cyclin D1^-^/_- _mouse embryonic fibroblasts, cyclins D1a and b have unique properties with regard to cell migration [33]. Cyclin D1a stabilizes p27^Kip1 ^and inhibits RhoA-induced ROCK kinase activity promoting cell migration while cyclin D1b fails to stabilize p27^Kip1 ^and has no effect on cell migration. Our results confirm that cyclin D1b does not affect LP-1 cells migration. Although cyclin K resembles cyclin D1a in agreement with its known biological functions: binding to CDK4/6, phosphorylation of pRb; one prominent feature of its structure is the impairment of p27^Kip1 ^binding [34]. Accordingly, cyclin K expression in LP-1 is associated with the absence of p27^Kip1^, the lack of migration capacity and an enhanced clonogenic potential *in vitro*. Experiments assessing the metastatic potential of LP-1-derived cells *in vivo *are ongoing.

### Cyclin D1b stimulates neoangiogenesis

Cyclin K/D1b-expressing cells, grafted onto the CAM of chicken embryo, generate within a few days tumors whose vascularization is significantly different. Tumors obtained in *nude *mice after *s.c*. injection of LP-1-derived cells show the same different vascularization. Indeed, LP-1 MM cells overexpressing cyclin D1b markedly promote tumor angiogenesis. Cyclin D1 regulates vascular endothelial growth factor (VEGF) production and thereby, growth of vascular endothelial cells and tumor [35]. The inhibition of tumor growth after local injection of VEGF siRNA confirmed a major role of VEGF in tumor expesnion. This result was further reinforced by the use of VEGFR inhibitors which could target either the MM tumoral cells or their immediate environment. Cyclins D1b and K induce transcriptional activation/inhibition of proangiogenic/antiangiogenic factors. One striking difference between the two cell lines is the overexpression of *FGFR3 *in LP-1D1b cells. Activation of the fibroblast growth factor 3 (FGFR3) expressed by myeloma cells and its ligand FGF present in the mouse could sustain *in vivo *angiogenesis such as in the bone marrow milieu [36]. The expression of 402 angiogenesis-associated genes has been studied in a large series of patients with a MM or a MGUS (monoclonal gammopathy of undetermined significance), considered as the premalignant state of MM, MM cell lines and their normal counterparts [37]. This study concluded that aberrant expression of proangiogenic and downregulation of antiangiogenic genes occur in all MM patients. Interestingly for our purpose, we noted that three genes were silent in MGUS and expressed in MM, namely *IL6*, *FGF9 *and *FGFR3*. It is tempting to speculate that the expression of FGFR3 triggers premalignant cells to enter a malignant state as observed in our model.

### Cyclin D1b and cyclin K activate major actors of MM tumorigenesis

**Figure 7:**
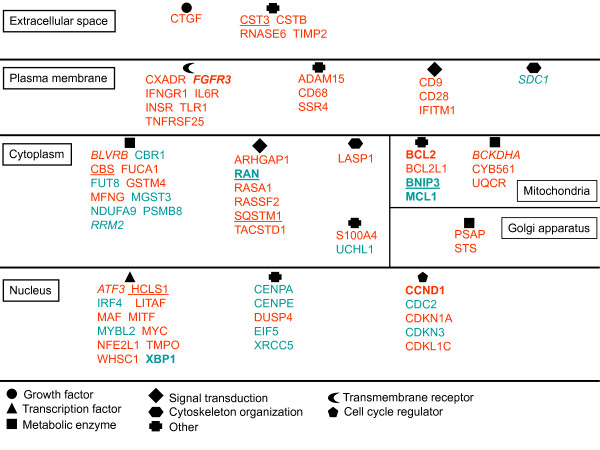
**Schematic representation of genes involved in the pathogenesis of MM**. We compared our microarray data with some previously published [37-41]. We then sorted genes characterized in our study and at least another one; genes cited in more than two studies are in bold. Genes are listed according to their subcellular localization and cellular function (symbols are explained under the scheme). Overexpressed genes are in red, underexpressed genes are in green. Genes altered by cyclin D1b expression are in italic, genes altered by both cyclins D1b and K are underlined.

Besides *CCND1*, several genes have been recognized as major actors of MM tumorigenesis: *CCND2*, *MAF*, *FGFR3*, *ITGB7 *and *CXCR3 *[13]. All of them are altered in either LP-1D1b or LP-1K cells. This observation validates the use of LP-1-derived cells as a paradigm of tumorigenesis in MM. Moreover, based on previous microarray analyses, genes implicated specifically in the tumorigenic process of MM have been characterized [38-42]. Several of them are also detected in our microarray analysis. They code for proteins involved in metabolism, signal transduction, transcription factors and cell cycle regulators (Figure 7). Among them only a few number of genes are recognized as tumorigenic in several MM models: *BCL2*, *BNIP3*, *FGFR3*, *MCL1*, *RAN *and *XBP1*. BCL2 protein is the archetype of apoptosis regulatory molecules; it is an integral outer mitochondrial membrane protein that blocks the apoptotic death. BCL2 is often overexpressed in transformed cells of the B lymphoid lineage, in malignant compared to normal plasma cells [39]. By contrast BNIP3 (BCL2/adenovirus E1B 19 kDa interacting protein 3) protein has pro-apoptotic function and *BNIP3 *gene is repressed in MM cells through the methylation of its promoter [42]. *MCL1 *encodes two proteins belonging to the BCL2 family with either pro- or antiapoptotic functions; its overexpression has been detected in blood sample from a myeloma patient but not in his twin [41]. The role of the transcription factor XPB1 and the nuclear protein RAN, a member of RAS family, in the myeloma pathogenesis remains to be defined. The t(4;14)(p16.3;q32) occurs in 15-20% of myeloma patients and leads to the overexpression of *FGFR3 *gene and, in turn, the constitutive activation of several signaling pathways in 80% of t(4;14)+ MM patients. Five genes (*ATF3*, *BCKDHA*, *FGFR3*, *RRM2 *and *SDC1*) are altered by cyclin D1b alone and six (*BNIP3*, *CBS*, *CST3*, *HCLS1*, *RAN*, *SQSTM1*) by both cyclins D1b and K. Those findings question the relevance of cyclin D1b expression in MM pathogenesis.

### Is cyclin D1b involved in MM pathogenesis?

We have previously shown that both isoforms of cyclin D1a and b mRNAs are present in MM cells and their relative levels similar. However, cyclin D1a isoform is predominant both in MM cell lines and primary cells [16]. It has been thought that *CCND1 *alternative splicing was regulated by a G/A polymorphism at the exon 4/intron 4 boundary [1]. It is now demonstrated that factors associated with chromatin remodelling and translation elongation largely contribute to cyclin D1b accumulation [43,44]. This indicates that the regulation of cyclin D1b level is complex and only the direct analysis of the cyclin D1b protein could define its impact on disease. In a recent large multiethnic case-control study, Knudsen and his group showed that cyclin D1b is clearly elevated in a significant fraction of primary breast tumors but with a heterogeneous level within specimens and underexpressed in asynchronously proliferating cell lines [45,46]. They also show unambiguously that cyclin D1b levels are associated with adverse prognostic outcome. Such an analysis of cyclin D1b protein level in MGUS, the primary step of MM and primary MM cells should be conducted in order to definitely conclude on its role in MM pathogenesis.

## Competing interests

The authors declare that they have no competing interests.

## Authors' contributions

VM and J-MR performed *in vivo *experiments; VM performed Q-PCR and IHC assays, GT performed western blots, confocal microscopy and flow cytometry; J-ML and JW-B performed CAM assays; DD and BJ performed microarray analyses and validated statistically the results; GA made computational analyses; VM, J-MR, JW-B, DD and BJ critically revised the manuscript; BS designed experiments, analyzed and interpreted the data, drafted the manuscript. All authors approved the final version of the manuscript and its submission for publication.

## Supplementary Material

Additional file 1 **Table S1**. Sequences of primers used for validation of microarray data by Q-PCR. Click here for file

Additional file 2 **Figure S1**. Hierarchical clustering of cyclin K-altered genes. Clustering was visualized with TigrMev 4_03 software http://www.tm4.org/mev.html. Sequences with FC>3 were selected. Expression levels are shown for either upregulated genes (red) or downregulated genes (green). The name of probes is indicated in the treeview. Click here for file

Additional file 3 **Figure S2**. Hierarchical clustering of cyclin D1b-altered genes. See legend of Additional File 2. Click here for file

Additional file 4 **Figure S3**. Hierarchical clustering of cyclin K- and D1b-altered genes. See legend of Additional File 2. Click here for file
