# Supplementary material for: Cyclin K and cyclin D1b are oncogenic in myeloma cells
Source: Mol Cancer. 2010 May 10;9:103. doi: 10.1186/1476-4598-9-103 (PMC2881116; doi:10.1186/1476-4598-9-103)
Supplement: Additional file 1 — Table S1. Sequences of primers used for validation of microarray data by Q-PCR. [file 1476-4598-9-103-S1.DOC]

**Table S1. Sequences of primers used for validation of microarray data by Q-PCR**

| **Gene** | **Forward (5’-3’)** | **Reverse (5’-3’)** |
| --- | --- | --- |
| *36B4* | gat gcc cag gga aga cag | tct gct ccc aca atg aaa cat |
| *CSN2* | ggt taa aca tga gga cca gca | gtg gct gga aag agg ggt a |
| *FGFR3* | gcc tcc tcg gag tcc ttg | cga aga cca act gct cct g |
| *FHIT* | tcc ctc cct ctg cct ttc | tct ctt ctt tcc tga gct tca aa |
| *HSP90B1* | ctg gaa atg agg aac taa cag tca | tct tct ctg gtc att cct aca cc |
| *TUBB2B* | agg acg gac aga ccc aga c | ctg atg acc tcc caa aac ttg |
| *TFRC* | ttg aga aaa caa tgc aaa atg tg | ccc agt tgc tgt cct gat ata ga |
| *CD48* | cct aca tca tga ggg tgt tga a | atg aca ggc ttg ggt aca gg |
| *LTB* | ccc agg atc agg gag gac | ggg ctg aga tct gtt tct gg |
| *FN1* | ctg gcc gaa aat aca ttg taa a | cca cag tcg ggt cag gag |
| *BCL2* | agt acc tga acc ggc acc t | ggc cgt aca gtt cca caa a |
| *CDK6* | ctt gtc cac atg gtg ctct c | ggc cgt aca gtt cca caa a |
| *GAPDH* | agc cac atc gct cag aca c | gcc caa tac gac caa atc c |
| *UCHL1* | cct gaa gac aga gca aaa tgc | aaa tgg aaa ttc acc ttg tca tct |
