# Supplementary figures and images for: Cyclin K and cyclin D1b are oncogenic in myeloma cells
Source: Mol Cancer. 2010 May 10;9:103. doi: 10.1186/1476-4598-9-103 (PMC2881116; doi:10.1186/1476-4598-9-103)

## Slide 1
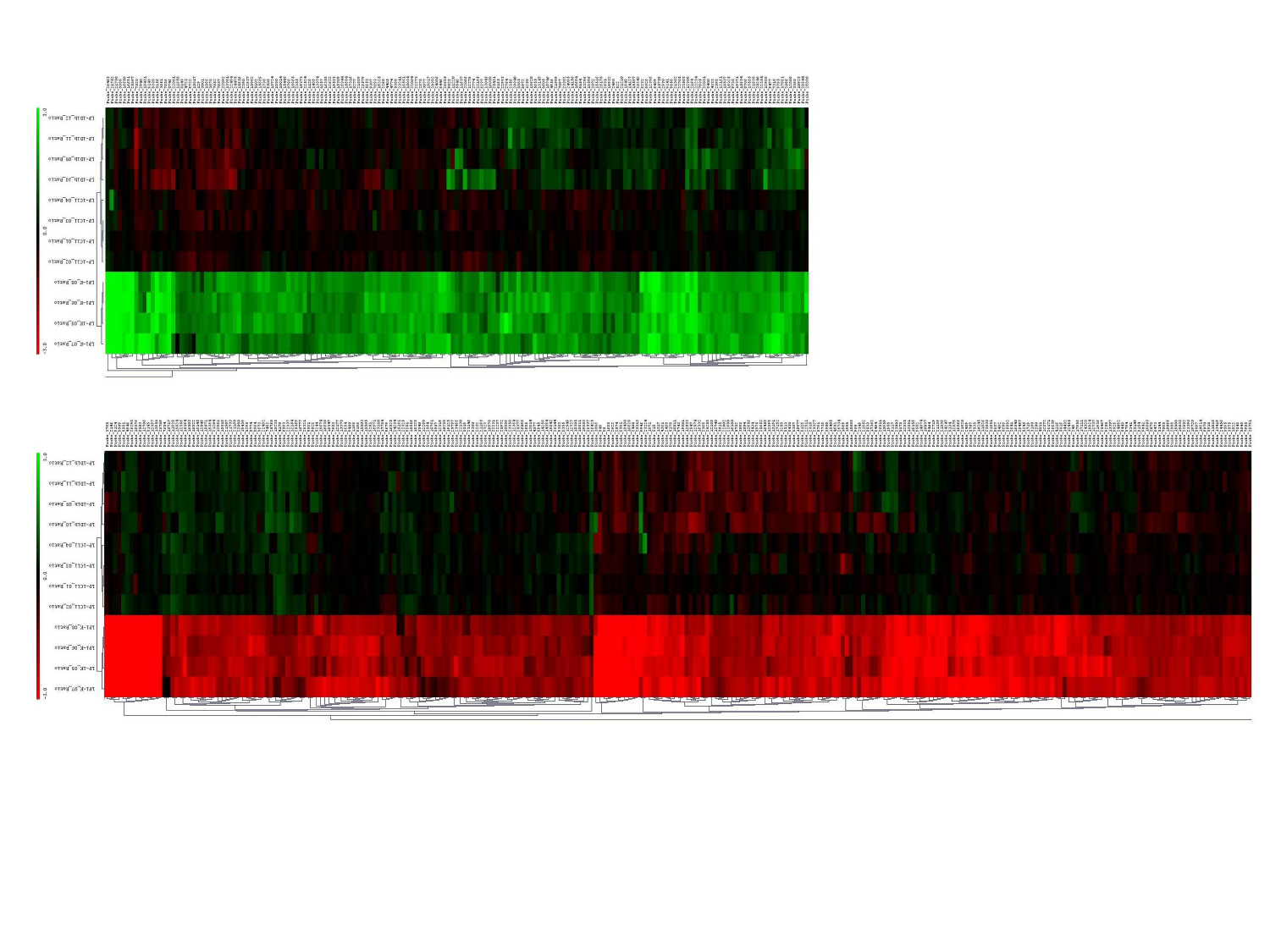

Supplement: Additional file 2 — Figure S1. Hierarchical clustering of cyclin K-altered genes. Clustering was visualized with TigrMev 4_03 software http://www.tm4.org/mev.html. Sequences with FC>3 were selected. Expression levels are shown for either upregulated genes (red) or downregulated genes (green). The name of probes is indicated in the treeview. [file 1476-4598-9-103-S2.PPT]

## Slide 1
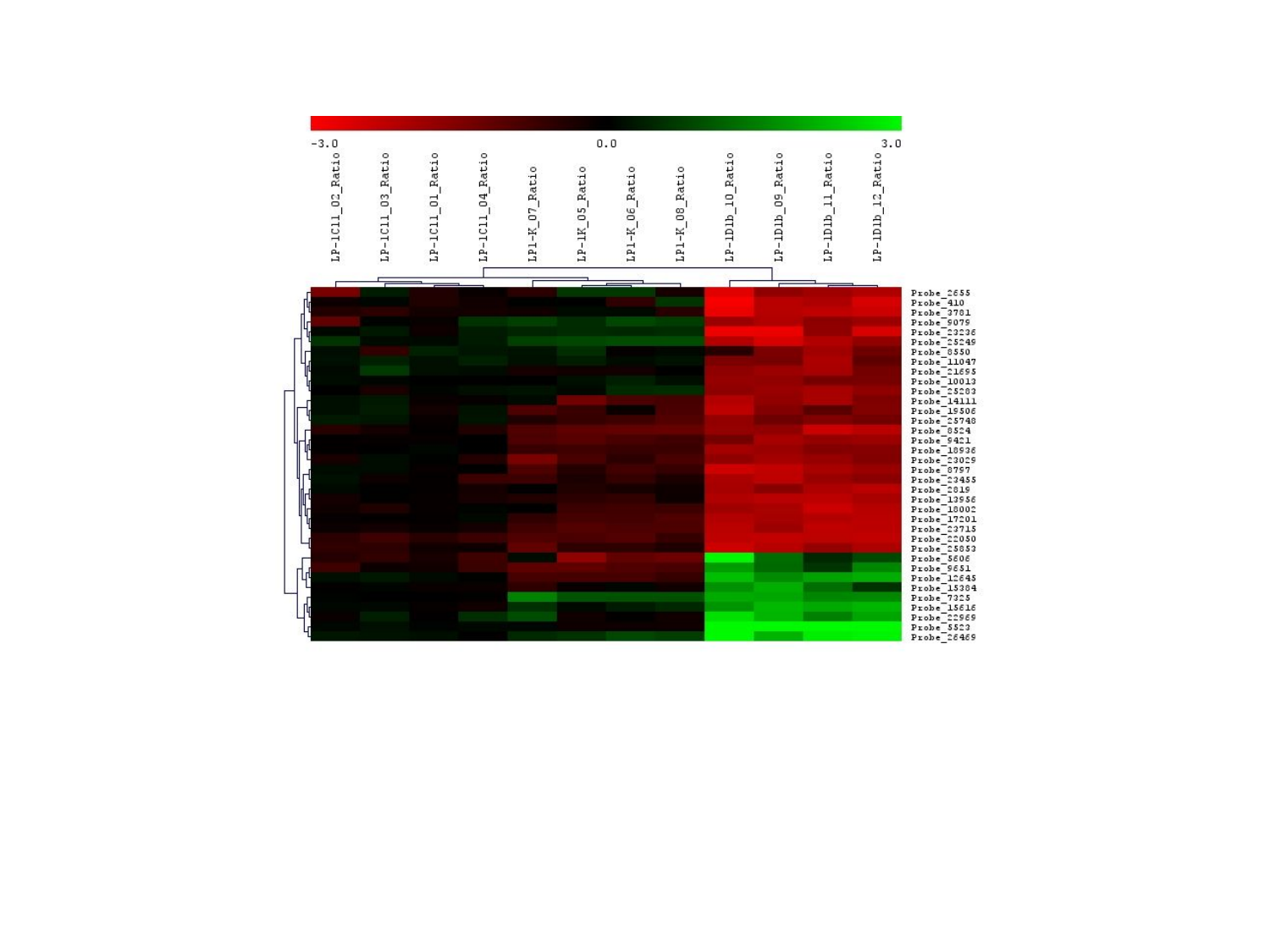

Supplement: Additional file 3 — Figure S2. Hierarchical clustering of cyclin D1b-altered genes. See legend of Additional File 2. [file 1476-4598-9-103-S3.PPT]

## Slide 1
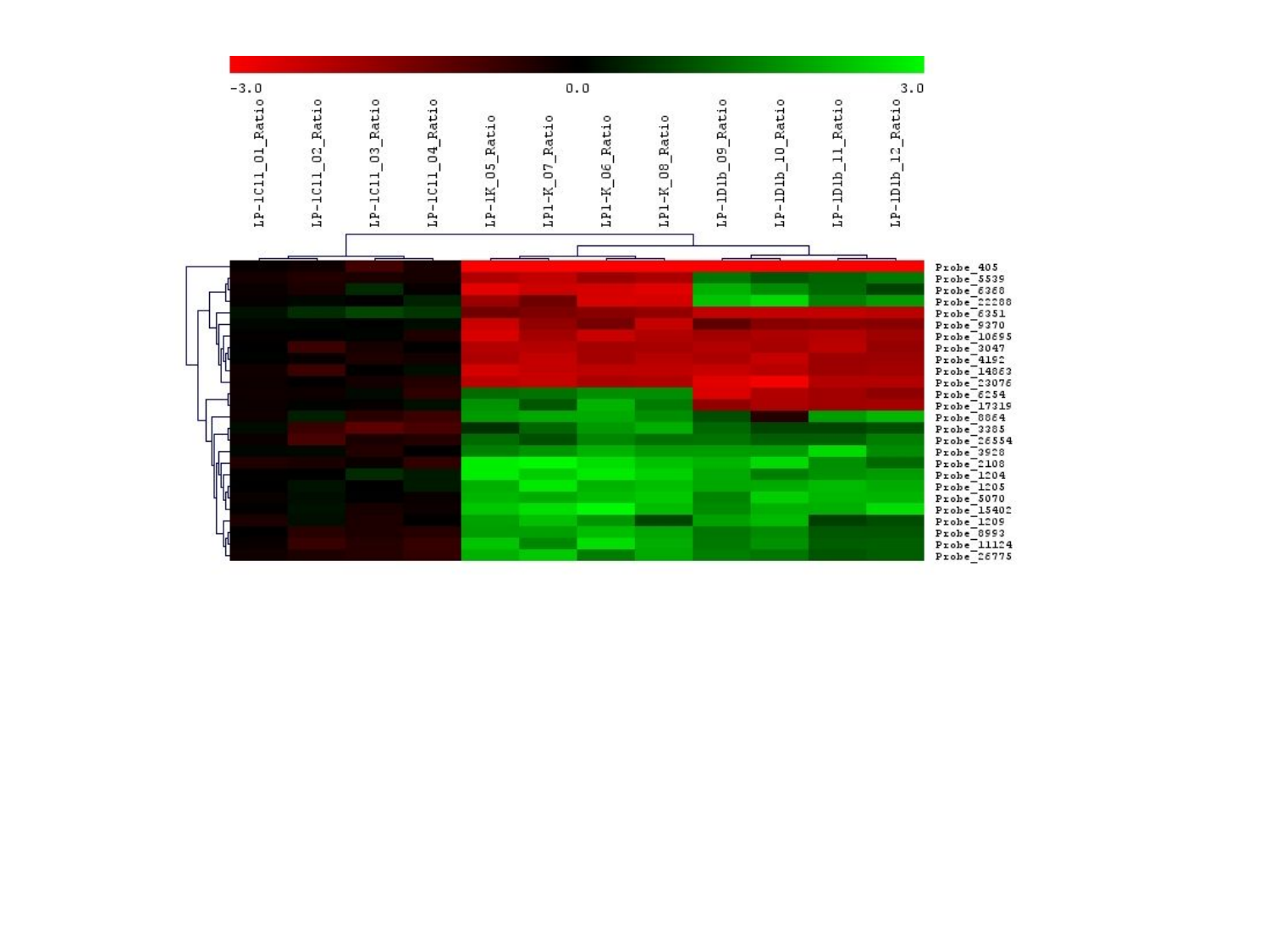

Supplement: Additional file 4 — Figure S3. Hierarchical clustering of cyclin K- and D1b-altered genes. See legend of Additional File 2. [file 1476-4598-9-103-S4.PPT]
